# DNA is preserved and maintains transforming potential after contact with brines of the deep anoxic hypersaline lakes of the Eastern Mediterranean Sea

**DOI:** 10.1186/1746-1448-4-10

**Published:** 2008-08-05

**Authors:** Sara Borin, Elena Crotti, Francesca Mapelli, Isabella Tamagnini, Cesare Corselli, Daniele Daffonchio

**Affiliations:** 1 University of Milan, Department of Food Science and Microbiology, Via Celoria 2, 20133 Milan, Italy; 2 University of Milan-Bicocca, Department of Geosciences and Geotechnologies, Milan, Italy

## Abstract

**Background:**

Extracellular dissolved DNA has been demonstrated to be present in many terrestrial and aquatic environments, actively secreted, or released by decaying cells. Free DNA has the genetic potential to be acquired by living competent cells by horizontal gene transfer mediated by natural transformation. The aim of this work is to study the persistence of extracellular DNA and its biological transforming activity in extreme environments like the deep hypersaline anoxic lakes of the Mediterranean Sea. The brine lakes are separated from the upper seawater by a steep chemocline inhabited by stratified prokaryotic networks, where cells sinking through the depth profile encounter increasing salinity values and osmotic stress.

**Results:**

Seven strains belonging to different taxonomic groups isolated from the seawater-brine interface of four hypersaline lakes were grown at medium salinity and then incubated in the brines. The osmotic stress induced the death of all the inoculated cells in variable time periods, between 2 hours and 144 days, depending on the type of brine rather than the taxonomic group of the strains, *i.e. Bacillaceae *or gamma-proteobacteria. The Discovery lake confirmed to be the most aggressive environment toward living cells. In all the brines and in deep seawater dissolved plasmid DNA was substantially preserved for a period of 32 days in axenic conditions. L'Atalante and Bannock brines induced a decrease of the supercoiled form up to 70 and 40% respectively; in the other brines only minor changes in plasmid conformation were observed. Plasmid DNA after incubation in the brines maintained the capacity to transform naturally competent cells of *Acinetobacter baylii *strain BD413.

**Conclusion:**

Free dissolved DNA is likely to be released by the lysis of cells induced by osmotic stress in the deep hypersaline anoxic lakes. Naked DNA was demonstrated to be preserved and biologically active in these extreme environments, and hence could constitute a genetic reservoir of traits acquirable by horizontal gene transfer.

## Background

Free extracellular DNA has been retrieved in many environments, both aquatic and terrestrial [1]. Naked DNA is actively excreted by growing cells, depending on biotic and abiotic factors [2-4], or is passively released in the environment by decaying cells. In hypersaline systems the water availability is reduced with the consequence of inferring water stress to cells. The osmotic stress in particular affects cell turgor and membrane integrity, leading to death by osmotic lysis of cells not adapted to hypersaline conditions [5], and in turn to the release of the cellular content, including nucleic acids.

Free dissolved DNA is used by microorganisms as source of N, P and C [6,7], and in the deep-sea environment has been hypothesised to constitute a key trophic resource, substantially contributing to P cycling [7]. Other than a nutrient source, dissolved DNA may have a genetic function as a source of genes acquirable by natural transformation. In terrestrial and aquatic environments several bacterial strains have been discovered to be naturally competent, *i.e. *to have the capacity to acquire exogenous naked DNA [4]. The acquisition of new genetic traits by horizontal gene transfer can constitute an evolutionary strategy for the selection of natural microbial communities [8]. In harsh and stressful conditions, in particular, gene exchange and rearrangements are estimated to increase in order to promote genome plasticity, increasing DNA repairing rates and evolutionary adaptation mechanisms [9,10].

In natural ecosystems the majority of extracellular DNA is converted in deoxyribose, inorganic orthophosphate, purines and pyrimidines by the enzymatic hydrolytic action of nucleases, present in most of the microbial habitats [11,12]. Besides the biological degradation, DNA is a chemically unstable molecule that decays spontaneously mainly through hydrolysis and oxidation [13]. Several physical and chemical factors can moreover compromise the integrity of naked DNA molecules once they are released by cells. Once free in the environment, DNA fragments are no longer preserved by cellular DNA repair mechanisms and, even if not severely degraded, they accumulate environmentally inflicted damages. Despite all these factors, extracellular DNA has been demonstrated to be preserved in soil, sediments, freshwater and seawater for different time periods [1], and even geologically ancient DNA has been retrieved from fossil materials [14,15]. Dell'Anno and Danovaro [7] estimated that the deep-sea sediments constitute the largest reservoir of extracellular DNA in the Earth's oceans, with 0.50 ± 0,22 Gt of extracellular DNA contained in the first 10 cm of sediments, and its residence time, resulted by the balance of release and degradation, is 9.5 years. In particular, in the sediments underlying the deep anoxic hypersaline lake l'Atalante Danovaro et al. [16] retrieved the highest concentration of extracellular DNA reported in a natural environment.

In hypersaline environments salt has been shown to have a stabilising effect on nucleic acids, protecting biological macromolecules against heat degradation [17,18]. On the other side, the reduced water activity of a salty environment has consequences on DNA conformation. The reduction of the hydration of the DNA molecules decreases the stabilisation of the structure that in high water activity environments is conferred by weakly bound water molecules [19].

The aim of this work is to study the persistence of extracellular DNA and its potential for gene exchange in extreme environments characterised by hypersaline and anoxic conditions. The deep hypersaline anoxic lakes of the Eastern Mediterranean Sea are unique deep-sea habitats originated from the dissolution of buried salt deposits emerging at the topography due to the strong faulting activity of the area. They are characterised by a salinity above 30%, absence of light, elevated pressure, variable pH values and ionic compositions. The sharp density difference between brines and normal sea water acts as a barrier, avoiding oxygen exchange, therefore the brines become oxygen-free and rich in hydrogen sulphide. Despite these harsh conditions, the brines that fill the lakes contain highly adapted active microbial communities [20]. The brines are separated from the upper seawater by a steep interface layer with salinity values ranging from seawater to the brine physio-chemical parameters. This layer is an enrichment phase for complex microbial networks rich in taxonomical and functional biodiversity that are stratified along the depth and salinity profile [21].

## Results and discussion

### Survival in the brines of bacteria isolated from the seawater-brine interface

**Figure 1:**
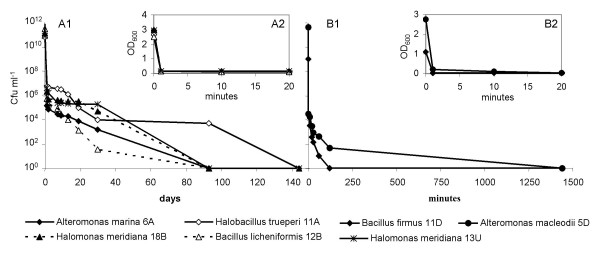
**Survival of bacterial cells in the brines.** Figure 1A1, 1B1: time series plate count quantification of different strains incubated in the brine of isolation, *i.e. *L'Atalante, Bannock, Urania (A1) and Discovery (B1). Note that the x-axis scale in B1 is in minutes, while in A1 is in days. Figure 1A2, 1B2: time series measurement of the optical density (OD_600_) of the strains incubated in the brines of L'Atalante, Bannock, Urania (A2) and Discovery (B2). Error bars are within the range of 0.3–71% of each value.

**Figure 2:**
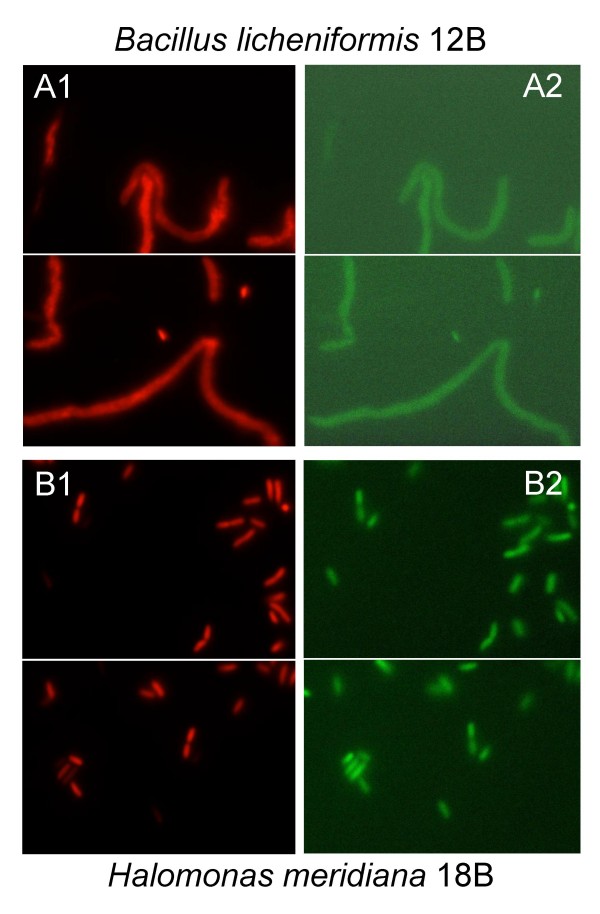
**Epifluorescence microscopy visualization of the strains *B. licheniformis *12B (A) and *H. meridiana *18B (B) after 10 minutes of incubation in the Bannock brines, stained with Propidium iodide (red) and SybrGreen (green). **A1, B1: visualisation at Propidium iodide excitation/emission wavelength (494/617 nm) of the cells with damaged cell wall. A2, B2: visualisation at SybrGreen I excitation/emission wavelength (494/519 nm) of the totality of the cells.

The interface between deep sea-water and the hypersaline anoxic brines is a thin layer of few meters over the brines that hosts high bacterial density and diversity [21]. Bacteria sinking through the interface encounter conditions of increasing salinity and, as a consequence, increasing osmotic stress. Seven strains have been selected, based on the following criteria: i) isolation from the seawater-brine interface of the four hypersaline lakes, L'Atalante, Bannock, Discovery, Urania, that have brines with very different chemical composition [20] and ii) belonging to different taxonomic groups, *i.e. *gamma-proteobacteria and high G+C sporeforming bacteria of the family *Bacillaceae *(table 1). The seven strains have been isolated from the less saline layers of the hypersaline basins, *i.e. *the seawater-brine interface, and do not exhibit halophilic features, being able to grow in absence of salt. They are moderately halotolerant, since all of them tolerate up to 10–12% of NaCl in the growth medium (table 1). Simulating a sink in the lower hypersaline layers of the lakes, the cells of the strains isolated from the different lakes were grown on medium with intermediate salinity (5%) and then incubated in the corresponding hypersaline brines. Figure 1(A1, B1) shows the dramatic decrease in cell viability over time of contact with the brines. All the strains are subjected to a strong stress immediately after the exposure to the brines, since the number of the viable cells decreased by 5–11 orders of magnitude in the first minute. The stress is osmotic and leads to cell lysis rather than just a loss of viability, as demonstrated by the parallel decrease in optical density of the suspension (figure 1, A2, B2). The majority of the cells that were not completely lysed, showed nevertheless damaged membranes after 10 minutes of incubation in the brines, as demonstrated by their staining with propidium iodide (figure 2).

**Table 1:** Strains used in the work, taxonomic identification and salinity tolerance

Strain name	lake of isolation^1)^	Medium of isolation	closest relative	homology %^2)^	NaCl tolerance (%)	
					min	max

6A	L'Atalante	DSMZ-246^3)^	*Alteromonas marina*	98	0	10
11A	L'Atalante	DSMZ-372	*Halobacillus trueperi*	100	0	10
12B	Bannock	Marine Broth (Difco 2216)^3)^	*Bacillus licheniformis*	98	0	12
18B	Bannock	DSMZ-246^3)^	*Halomonas meridiana*	99	0	10
5D	Discovery	DSMZ-246^3)^	*Alteromonas macleodii*	99	0	10
11D	Discovery	DSMZ-246	*Bacillus firmus*	99	0	10
13U	Urania	Marine Broth (Difco 2216)	*Halomonas meridiana*	99	0	10

^1) ^this is also the brine in which the strain has been incubated for the survival experiments

^2) ^the strains have been identified based on the percentage homology on the 16S rRNA sequence with the sequences contained in public databases

^3) ^medium diluted 1:10 with sterile seawater

The rate of viability loss seems to depend on the type of brine rather than type of microorganism. In the Discovery brine both the strains tested, *Bacillus firmus *11D and *Alteromonas macleodii *5D, did not show any remnant living cell after only 2 and 24 hours of incubation respectively. When compared with the other 3 brines, the Discovery one is indeed characterised by the most extreme composition, with a concentration of MgCl_2 _that reaches 5 M [20], and has been shown to be highly hostile toward life [22]. In the other brines all the strains exhibited longer survival times, demonstrating viable cells up to 30–93 days, depending on the strains. *Halobacillus trueperi *11A was the most resistant of all the strains tested, showing the complete disappearance of viable cells after 144 days of incubation in L'Atalante brines. Further experiments of cross inoculations would confirm whether the various degrees of lethal effect of the brines depend only by their different composition, or could be related to the peculiar physiological features of the microbes colonising each lake.

Based on these results it is possible to hypothesise that, as a consequence of the osmotic stress encountered during sinking through the depth profile of the basins, cells not adapted to hypersalinity decay releasing their cellular content, including nucleic acids, during osmotic lysis. The following part of the work aims to understand the fate of naked DNA once released from the decaying cells.

### Survival in the brines of plasmid DNA and transformation potential

**Figure 3:**
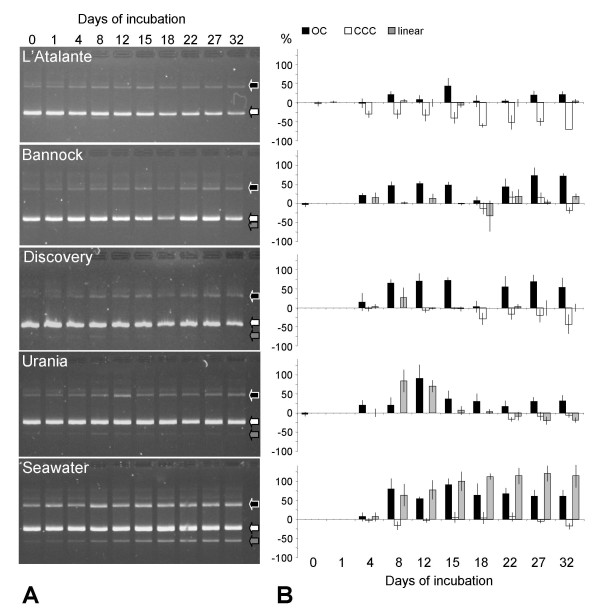
**Stability of plasmid DNA in the brines and seawater.** A: gel electrophoresis of pZR80(gfp) incubated for increasing periods of time in brines and seawater. B: relative quantification of the different plasmid conformations, OC (open circular), linear, CCC (covalently closed circular).

Figure 3A indicates the gel electrophoresis pictures showing the fate of the plasmid DNA during a 32 day-long incubation, whereas Figure 3B shows the relative quantification of the main plasmid bands as a mean of different experiments. In all the incubation experiments the DNA proved to be highly preserved, and the total plasmid quantity did not show remarkable degradation for the first 15 days of incubation (p < 0.01 in L'Atalante, Urania, Discovery brines). This result confirms previous findings by DeFlaun and Paul [23] who performed short term experiments of 36 hours incubation using sterile seawater. In non sterile conditions extracellular DNA is degraded in few hours both in seawater and in freshwater [23,24], due to enzymatic DNA degradation [1]. In this work the preservation of dissolved DNA has been studied in 0.22 μm pore size filtered systems, in order to selectively investigate the effects of the four anoxic brines with different ionic composition without the interference of cells that are retained on the filter. For the first 3 days of incubation brines or seawater did not show any apparent effect on DNA. After this period in the L'Atalante brine the supercoiled conformation of the plasmid (CCC form) decreased up to 70 ± 15%. The Discovery brine, which was the most aggressive toward living cells, induced a decrease up to 43 ± 25% of the CCC form between 18 and 32 days of incubation. The exposure to Urania and Bannock brines as well as the seawater did not severely affect the total quantity of DNA over the 32 days of the experiment, but mainly affected the conformation of the plasmid molecule. Except for incubation in L'Atalante brines, the CCC form decrease was minor, between 0 and 29 ± 16%, while the other forms increased in variable percentages when compared with the respective bands at the beginning of the experiment (figure 3). The incubation of pZR80(gfp) plasmid in seawater or Urania brine lead to the appearance after 8 days of a DNA band with higher electrophoretic mobility than the supercoiled form, that could be attributed to the linear plasmid. The results showed that seawater or hypersaline brines in anoxic conditions in the absence of cells and suspended material retained on 0.22 μm filters had a partial, in L'Atalante and Discovery brines, or negligible, in the other cases, degradative effect on the overall DNA molecules. This could be due to a nicking effect on the plasmid DNA molecules, leading to the opening of the supercoiled form toward more relaxed conformations. These high values of DNA preservation confirm previous findings in sediments underlying the brines of the L'Atalante lake, which reported high number of spores [25], exceptionally high concentrations of extracellular DNA [16], and in general a high level of organic matter preservation [26].

**Figure 4:**
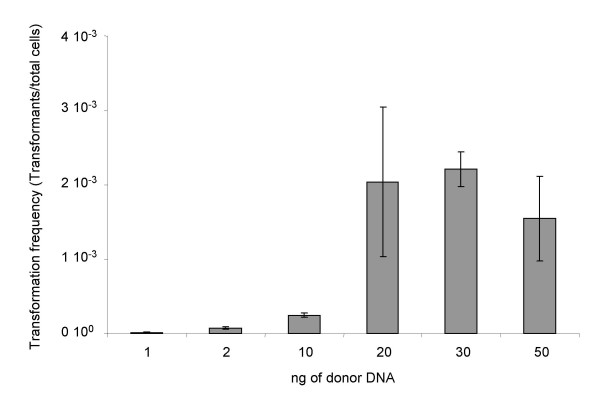
Frequency of transformation of naturally competent cells of *A. baylii *BD413 with different quantities of plasmid pZR80(gfp).

**Figure 5:**
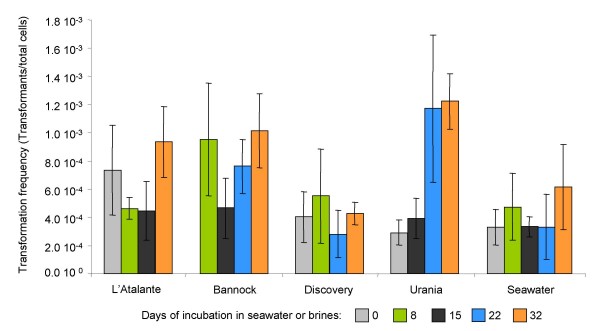
Frequency of transformation of naturally competent cells of *A. baylii *BD413 with plasmid pZR80(gfp) incubated for different time periods in brines and seawater.

To investigate the biological effect of the degradation or changes in conformation induced by brines or seawater, we tested the efficiency of the rescued plasmids in transformation. Plasmid pZR80(gfp) recovered after incubation in seawater and brines was used to transform naturally competent cells of *A. baylii *BD413, and the efficiency of transformation was calculated. To exclude that differences in transformation efficiency were due to differences in quantity of the donor DNA rather than in its quality, we estimated the minimum quantity of donor DNA that did not affect the transformation efficiency. The results demonstrated that applying between 20 and 50 ng of donor DNA per transformation assay did not significantly alter the transformation frequency (p < 0.03), giving an average of 1.9 ± 0.4 × 10^-3 ^transformants/total cells (figure 4). Based on these data, the transformation efficiency of pZR80(gfp) incubated in seawater or brines was calculated using equal concentrations of 25 ng of donor DNA per assay. The results (figure 5) showed that the incubation in all the four brines or in seawater did not affect the biological activity of extracellular dissolved plasmid DNA. pZR80(gfp) maintained similar values of transformation frequency for 32 days, with an average value of 5.6 ± 3.1 10^-4 ^transformants/total cells. Transformation frequency was on average lower than that calculated using pure plasmid extracts, probably due to incomplete desalting of plasmid preparations recovered from seawater and brines. Brines of the Urania basin that showed negligible degrading effects on dissolved DNA (figure 3), induced nevertheless a significant (p = 0.037) increase in transformation frequency after 22 days of incubation from 4.9 ± 0.3 × 10^-4 ^to 1.2 ± 0.2 × 10^-3^. This result could be due to a kind of effect at molecular level induced by the brines on the dissolved DNA, that was not visible by agarose gel electrophoresis and should be further explored. The dependence of the frequency of transformation upon the topological form of the plasmid has been described [27].

The integrity of the genetic information acquired by horizontal gene transfer was confirmed by evaluating the expression of the *gfp *gene which codifies for a green fluorescent protein that conferred fluorescent phenotype to the transformants (*data not shown*).

## Conclusion

At the interface between deep seawater and brine in the hypersaline anoxic lakes of the Eastern Mediterranean Sea bacterial communities are sharply stratified according to the increasing levels of salinity. The process of particulate sinking throughout the depth profile along the interface could expose non-adapted bacteria to higher salinity, with consequent osmotic stress that induces the lysis of the cells thereby releasing nucleic acids. With this work we demonstrated that this process is likely to occur in brines with different chemical composition, the released DNA is preserved biologically active in these extreme environments as in seawater, and the preserved DNA retains its transforming potential. Even though the role 0.22 μm filterable agents like DNAses, viruses or ultramicrobacteria should be further evaluated to estimate biological degradation of extracellular DNA, and the presence of naturally competent bacteria should be demonstrated, this work gives a first insight on the potential role of dissolved extracellular DNA in hypersaline environments to constitute a reservoir of genetic information that can be acquired by horizontal gene transfer mediated by natural transformation.

## Methods

### Brines and bacterial strains

Brines used in this study were recovered in 2003 from the deep submarine lakes Urania, Bannock, Discovery, L'Atalante located in the Eastern Mediterranean Sea, during a cruise of the R/V Urania [20]. Brines were sterilised by filtration on 0.22 μm pore size filters immediately after recovery, and then filtered a second time before the survival/preservation experiments.

Strains used in the work were isolated from the seawater/brine interface of each basin [21] and maintained in Plate Count Broth (PCB) (Difco, Milan, Italy) supplemented with 5% NaCl (PCB5%). The strains have been identified by sequencing of the PCR-amplified 16S rRNA gene and comparison of the sequence with public databases [21]. The list of the strains and the isolation media are reported in table 1.

### Survival of bacterial cells in the brines

Cells were harvested by centrifugation from 10 ml of overnight cultures incubated at 28°C in the medium PCB5%. Cells were washed with sterile NaCl solution (5%), and then resuspended in 1 ml of the same solution. One hundred μl of the bacterial suspension were inoculated in 10 ml of filter sterilised brine and incubated at 15°C, the *in situ *temperature of the hypersaline anoxic lakes. At defined time intervals, up to 140 days (figure 1, A1, B1), 100 μl of the incubated suspension was collected, immediately subjected to serial dilutions in NaCl solution (5%) and plate counted on agarised PCB5% in triplicate. At the same time intervals (figure 1, A2, B2) the optical density at 600 nm wavelength (OD_600_) of an aliquot of the suspension was analysed in a spectrophotometer (Beckman, DU640).

### Preservation of plasmid DNA in the brines

To simulate the release of DNA from the decaying cells, known amounts of purified plasmid pZR80(gfp) were incubated in the filtered sterile brines of the four basins or, as a control, in deep-sea water. The incubations have been carried out simulating the *in situ *conditions, at 15°C in the dark and in the absence of oxygen. At selected time intervals triplicate aliquots of the plasmid-containing solution were collected and the relative abundance of the different plasmid conformations was analysed by gel electrophoresis. Plasmid pZR80(gfp), used in this study, was constructed by the insertion of a gfp-cassette coding for a Green fluorescent protein in the plasmid pZR80-2 [28]. Briefly, the 1.1-kb gfp-cassette was amplified by standard PCR by using p*PnptII::gfp *plasmid as template [29] and primers PnptII1F-SphI (5'-ATTATTGCATGCAACCGGAATTGCCAGCT-3') and TendR-SphI (5'-ATTATTGCATGCCCAATTCCTGGCAGTTTATG-3'), both containing a SphI restriction site (underlined) with 5' overhang. The SphI digested PCR product was ligated in the SphI linearized pZR80-2 plasmid and used to transform competent *E. coli *cells.

Plasmid pZR80(gfp) was extracted from an overnight culture of the strain *E. coli *(pZR80(gfp)) with the QuiaPrep Mini Kit (Quiagen, Milan, Italy), quantified determining the optical density at 260 nm wavelength in a spectrophotometer (Beckman DU640). 212 μl of the plasmid preparation was inoculated to 10 ml of anoxic sterile filtered brines (final concentration 2 μg ml^-1^), and incubated in the dark at 15°C. At defined time intervals (figure 3) up to 32 days, a 100 μl aliquot of the plasmid containing brines were collected. All the inoculation and collection operations were conducted in an anoxic glove box. The recovered plasmid was desalted by dialysis against sterile milliQ water on a floating 0.1 μm pore size filter (25 mm diameter, Millipore, Milan, Italy) and stored at -20°C. At the end of the time course experiment 10 μl of each desalted aliquots were visualized by agarose gel electrophoresis in 0.5× TBE buffer stained with ethidium bromide. The relative abundance of the different plasmid conformations were estimated from the brightness of the plasmid bands in the electrophoresis gel picture using the software QuantityOne (Bio-Rad, Milan, Italy).

### Transformability of DNA after incubation into the brines

Desalted plasmid preparations recovered after incubation in the brines were used as donor DNA for the transformation of naturally competent cells of *Acinetobacter baylii *strain BD413, as described by Rizzi et al. [30]. Briefly, bacterial cells of *A. baylii *BD413 were recovered by centrifugation of 50 ml of an overnight culture in LB medium (Amersham Biotech, Milano, Italy), washed in sterile saline solution (NaCl 9 g l^-1^), resuspended in 10 ml of saline solution containing 15% (V/V) glycerol, divided in 100 μl aliquots and stored at -80°C. Cell concentration in each aliquot was 3.4 ± 0.8 × 10^8 ^cfu/ml. Sixteen μl of donor plasmid DNA were mixed with a thawed aliquot of naturally competent *A. baylii *cells, placed on a sterile mixed cellulose esters 0.22 μm pore size filter (47 mm diameter, Millipore, Milan, Italy), and positioned on the surface of an LB agar plate. After overnight incubation, the cells were recovered from the filter by resuspension in 5 ml of saline solution, serially diluted and plated on Luria Bertani (LB) (Amersham Biotech, Milan Italy) agar to count the total number of cells present on the filter, and LB agar supplemented with 100 μg/ml kanamycin to selectively count the transformant cells that acquired the plasmid. Transformation frequency was calculated as the number of Km^R ^transformants over the total number of cells. Results are an average of triplicate experiments. Randomly selected colonies were checked for the expression of the *gfp *gene contained in the pZR80(gfp) plasmid, coding for a Green fluorescent protein, by epifluorescence microscopy (Zeiss Axioplan).

### Live/dead staining

Cells from 200 μl of overnight cultures in medium PCB5% were collected by centrifugation (10 minutes 10000 *g*) and resuspended in 50 μl of sterile NaCl solution (5%). Five hundred μl of brine was added to the suspension. After 10 minutes of incubation 10 μl of the suspension was diluted with 100 μl of sterile water, and added with 3 μl of SybrGreen and 3 μl of propidium iodide solutions following the manufacturers instructions (Live/Dead staining kit, Molecular Probes). After 20 minutes of incubation at room temperature in the dark, the suspension was filtered on black polycarbonate filters (Millipore, Milan, Italy) and observed in epifluorescence (Zeiss Axioplan).

## Competing interests

The authors declare that they have no competing interests.

## Authors' contributions

SB designed and coordinated the work and wrote the manuscript. EC carried out the incubation and transformation experiments. FM carried out extracellular DNA quantifications. IT participated in microscopy analyses. CC was responsible of sample collection and at-sea operations. DD participated in conceiving and designing the work and revised the manuscript. All authors read and approved the final manuscript.
